# Decreased CCL5 expression in endometrial stromal cells induces deficient CCR5 ^+^CD4 ^+^ T cells in endometriosis: Deficient CCR5 ^+^CD4 ^+^ T cells in endometriosis

**DOI:** 10.3724/abbs.2024178

**Published:** 2025-01-07

**Authors:** Yue Li, Yunyun Li, Yewei Lu, Yikong Lin, Xiaolin Wang, Yizhun Zhu, Qiongjing Zeng, Meirong Du

**Affiliations:** 1 State Key Laboratory of Quality Research in Chinese Medicine and School of Pharmacy Macau University of Science and Technology Macau SAR China; 2 Department of Obstetrics and Gynecology Shanghai Fourth People’s Hospital School of Medicine Tongji University Shanghai 200092 China; 3 Laboratory of Reproduction Immunology Shanghai Key Laboratory of Female Reproductive Endocrine Related Diseases Obstetrics and Gynecology Hospital and Institute Fudan University Shanghai Medical College Shanghai 200433 China; 4 Department of Reproductive Medical Center West China Second University Hospital Sichuan University Chengdu 610065 China; 5 Key Laboratory of Birth Defects and Related Diseases of Women and Children (Sichuan University) Ministry of Education Sichuan University Chengdu 610065 China

**Keywords:** endometriosis, ectopic endometrial stromal cell, CCR5 ^+^CD4 ^+^ T cell, CCL5

## Abstract

Endometriosis (EMS) is a benign gynecological disease characterized by the growth of endometrial tissue outside the uterine cavity. Evidence shows that the survival of patients with ectopic endometrial implants is associated with a dysregulated immune microenvironment. CD4 ^+^ T cells can regulate EMS through diverse cytokines, the inflammatory response, and angiogenesis. CCR5 ^+^CD4 ^+^ T cells exhibit increased cellular immunogenicity and play a role in infectious diseases, host defense, and cancer progression. However, the specific mechanisms of CCR5 ^+^CD4 ^+^ T cells in EMS remain unknown. In the present study, flow cytometry and RNA-seq are utilized to assess the proportions and features of CCR5 ^+^CD4 ^+^ T cells in EMS patients, RT-PCR and ELISA are used to assess the production of CCL5 by ectopic endometrial stromal cells (ecESCs). Two EMS models are established through C57B6 wild-type and CCL5 ^‒/‒^ mice and utilized to explore the *in vivo* effects of CCR5 ^+^CD4 ^+^ T cells on ectopic lesions. Compared with CCR5 ^‒^CD4 ^+^ T cells, CCR5 ^+^CD4 ^+^ T cells display a more activated and cytotoxic phenotype. Diminished CCR5 ^+^CD4 ^+^ T cells and their impaired ability to produce IFN-γ are observed in the ectopic lesions of EMS patients and in murine EMS models. Impaired production of CCL5 has been detected in human ecESCs. Moreover, endometria stripped from CCL5 ^‒/‒^ mice are more likely to generate ectopic lesions in the peritoneum of recipient mice. These findings demonstrate that the attenuated recruitment of CCR5 ^+^CD4 ^+^ T cells in ectopic lesions caused by decreased production of CCL5 in ecESCs may facilitate the progression of EMS.

## Introduction

Endometriosis (EMS) is a prevalent, chronic, and painful gynecological disease in which active endometrial tissue grows outside the uterine cavity. It affects approximately 15% of women and negatively impacts their quality of life (such as dysmenorrhea, dyspareunia, and chronic pelvic pain) [ 1‒ 3] . Available treatment options for EMS are limited, and the recurrence rate is alarmingly high [4]. Therefore, it is necessary to investigate the pathogenesis of EMS. Menstrual reflux is the most accepted theory used to explain the development of EMS. However, this hypothesis cannot fully explain the cause of EMS [5]. Other factors, such as stem cells, immunological factors, and epigenetics, might play a part in EMS development.

A disorganized local immune microenvironment is a pivotal promoter of EMS. The abnormal activation of T cells [6], weakened phagocytic ability of macrophages [ 7, 8] , and decreased cytotoxicity of NK cells [9] contribute to the growth of ectopic endometrial implants. Accumulating evidence has shown that systemic and local variations in CD4 ^+^  T cells are closely related to the pathogenesis and development of EMS [ 10‒ 12] .

Some studies have shown that there is no significant difference in the proportion of CD4 ^+^ T cells in the peritoneal fluid between healthy women and EMS patients [13], whereas other studies have reported increased numbers of peritoneal CD4 ^+^ T cells among EMS patients [ 14, 15] . Increased numbers of peritoneal Tregs, which can promote the growth and invasion of ectopic lesions via the induction of macrophage polarization and the production of profibrotic cytokines, are found in EMS patients [ 16, 17] . Increased numbers of peritoneal Th17 cells in EMS patients secrete IL-17A, which promotes the inflammatory response and proliferation of ectopic endometrial stromal cells [18]. Peritoneal CD4 ^+^ T cells in EMS patients show Th2 predominance, and Th2 cells secrete IL-4 and IL-13 to promote fibrosis by mediating the differentiation of fibroblasts into myofibroblasts in ectopic lesions [19].

CCR5 ^+^CD4 ^+^ T cells are abundant in the endometrium, and CD38 ^+^HLA-DR ^+^ CCR5 ^+^CD4 ^+^ T cells exhibit an immune-activating phenotype [20]. CCR5 ^+^CD4 ^+^ T cells can increase CD40L/CD40-mediated APC maturation and promote the activation of DCs and CD8 ^+^ T cells, thus playing an anti-tumor role [21]. However, whether CCR5 ^+^CD4 ^+^ T cells play a role in the development of EMS has not been studied.

In this study, we focused on whether there is insufficient infiltration of CCR5 ^+^CD4 ^+^ T cells in ectopic lesions of EMS patients. Next, we analyzed the characteristics of focal CCR5 ^+^CD4 ^+^ T cells, compared the differences between CCR5 ^+^CD4 ^+^ T cells and CCR5 ^‒^CD4 ^+^ T cells, and explored the potential recruitment of CCL5 from ecESCs. Finally, we confirmed that impaired CCL5 production in ecESCs can lead to decreased CCR5 ^+^CD4 ^+^ T cells in ectopic lesions, thus promoting EMS development.

## Materials and Methods

### Human sample collection

Samples were collected from patients at the Obstetrics and Gynecology Hospital Affiliated with Fudan University between 2021 and 2023. The inclusion criteria for non-EMS patients were as follows: 18‒48 years old, no menopause, no malignant lesions, and no hormone therapy within the past 3 months. The inclusion criteria for EMS patients were as follows: 18‒48 years old, non-menopausal, diagnosed with EMS through laparoscopy, no adenomyosis, no uterine leiomyoma, no polycystic ovary syndrome, no abdominal inflammatory diseases or malignancy, and no hormone therapy within the past 3 months. The peripheral blood mononuclear cells (PBMCs) of 30 non-EMS patients, the PBMCs of 30 EMS patients, the peritoneal fluid (PF) of 30 non-EMS patients, the PF of 30 EMS patients, 50 eutopic endometria of healthy women (NuE), and 35 ectopic endometria of EMS patients (EcE) were collected. Endometrial tissues from non-EMS women were collected through hysteroscopy or diagnostic curettage, whereas PFs were collected through uterine laparoscopy. Endometrial tissues were collected through hysteroscopy or diagnostic curettage, whereas PF and ovarian endometriosis lesions were collected from EMS patients through uterine laparoscopy. The collection and use of these samples were approved by the Human Research Ethics Committee of the Obstetrics and Gynecology Hospital of Fudan University (Shanghai, China), and informed consent was obtained from each patient.

### Isolation and culture of endometrial stromal cells

The tissues were cut into 2- to 3-mm pieces after the blood was removed with PBS. The tissue fragments were digested in PBS containing 0.1% type IV collagenase (17104019; Gibco, Carlsbad, USA) at 37°C for 30‒60 min. The digested cells were filtered through 100-, 200-, and 400-mesh wire screens to remove undigested tissue lumps that may contain glandular epithelium. After filtration, the cells were centrifuged at 300 *g* for 5 min, and the supernatant was discarded. The cells were subsequently resuspended in DMEM/F-12 (SH30023.01; HyClone, Carlsbad, USA) supplemented with 10% fetal bovine serum (FBS; F8318; Sigma, St Louis, USA) and 0.5% triple antibiotics (E607063; Sangon, Shanghai, China) at 37°C in 5% CO _2_.

### Flow cytometry

Cell surface and intracellular molecular expressions were evaluated by flow cytometry using the CytoFlex flow cytometer (Beckman Coulter, Pasadena, USA). Fluorescein-conjugated anti-human antibodies, including CD3 (FITC), CD4 (BV510), CCR5 (PE), IL-4 (APC), IL-17A (BV421), INF-γ (BV605) (isotype ctrl: FITC Mouse lgG2a, BV510 Rat lgG2a, PE Rat lgG2b, APC Rat lgG1, BV421 Mouse lgG1, BV605 Mouse lgG1), and anti-mouse antibodies, including CD3 (FITC), CD4 (BV510), and CCR5 (APC) (isotype ctrl: FITC Rat lgG2b, BV510 Rat lgG2a, APC Armenian Hamster lgG) (BioLegend, San Diego, USA), were used according to the manufacturer’s procedures. For cell-surface staining, single-cell suspensions were stained on ice for 30 min in PBS supplemented with 1% antibodies. To detect intracellular cytokines, the cells were stimulated for 4 h with a cell activation cocktail (with brefeldin A) (#423303; BioLegend). Then, the cells were harvested, stained for surface expression, fixed and permeabilized for intracellular staining with a Fix/Perm kit (#421002; BioLegend). Flow cytometry data were analyzed using FlowJo software.

### ELISA

The PF of healthy women and EMS patients, the cell supernatant of normal eutopic endometrial stromal cells from normal patients (nuESCs), and ectopic endometrial stromal cells from EMS patients (ecESCs) were collected to evaluate the levels of CCL5. The concentration of CCL5 was measured using an ELISA kit (EK1129; MultiSciences, Shanghai, China) according to the manufacturer’s instructions.

### Quantitative real-time PCR

Total RNA was extracted using RNA purification kit (B0004D; EZB, Boston, USA) and then reverse-transcribed into first-strand complementary DNA (cDNA) via Hifair III 1st Strand cDNA Synthesis SuperMix for qPCR (#11141; YEASEN, Shanghai, China) according to the manufacturer’s instructions. The synthesized cDNA was amplified with specific primers and SYBR Green (#11202ES03; YEASEN) using an ABI PRISM 7900 Sequence Detection System (Applied Biosystems, Foster City, USA). Triplicate samples were examined for each condition. A comparative threshold cycle value for each sample was normalized via the 2 ^–ΔΔCt^ method.

### Isolation of CCR5 ^+^CD4 ^+^ T cells and CCR5 ^‒^CD4 ^+^ T cells

PBMCs isolated from 3 healthy women were separated via gradient centrifugation using Lymphoprep (#07851; STEMCELL, Cambridge, USA). CD4 ^+^ T cells were obtained by positive selection with a CD4 ^+^ T-cell isolation kit (#130-096-533; Miltenyi, Bergisch Gladbach, Germany). Then, according to the instructions for magnetic bead separation (#359105; BioLegend; #130-048-801; Miltenyi), the cells captured by the magnetic column were quickly flushed into a new centrifuge tube to obtain CCR5 ^+^CD4 ^+^ T cells, and the cell suspension flowing through the MS column was composed of CCR5 ^‒^CD4 ^+^ T cells.

### RNA-sequencing analysis

Total RNA from CCR5 ^+^CD4 ^+^ T cells and CCR5 ^‒^CD4 ^+^ T cells isolated from the PBMCs of 3 healthy women were isolated using Trizol reagent (Invitrogen, Carlsbad, USA). The libraries were constructed using the VAHTS Universal V6 RNA-seq Library Prep Kit (NR604-01; Vazyme, Nanjing, China) according to the manufacturer’s instructions. Transcriptome sequencing and analysis were conducted by OE Biotech Co., Ltd. (Shanghai, China). We considered transcripts to be differentially expressed if the *P* value was less than 0.05 and if the fold change was either > 1 or < ‒1. On the basis of the hypergeometric distribution, GO, KEGG, and gene set enrichment analyses (GSEAs) of the DEGs were performed to screen the significantly enriched terms using R (v 3.2.0). R (v 3.2.0) was used to construct the column diagram and heatmap of the significantly enriched terms.

### Mouse EMS model

After two weeks of adaptation, the mice were randomly chosen as donors for the EMS model. The donor mice were intraperitoneally injected with E2 at a dosage of 0.2 μg/g body weight three times per week. Mice in oestrus were selected through vaginal smears as receptors for the EMS rat model. The endometrium of the donor mouse was removed. The stripped endometrium was sutured to the peritoneum of the recipient mice. After 2 weeks, the recipient mice were euthanized with CO _2_. The endometrium, ectopic endometrium, and peritoneal fluid of the recipient mice were collected for subsequent studies.

### Statistical analysis

GraphPad Prism 8 was used to analyze the experimental results. Student’s *t* test was used to compare the statistical significance of the differences between two groups, and one-way analysis of variance (ANOVA) was used to compare the statistical significance of the differences among multiple groups. The statistical results are expressed as the mean ± SEM, and *P*  < 0.05 indicated statistical significance.

## Results

### Reduced infiltration of CCR5 ^+^CD4 ^+^ T cells in ectopic lesions from patients with EMS

**Figure 1:**
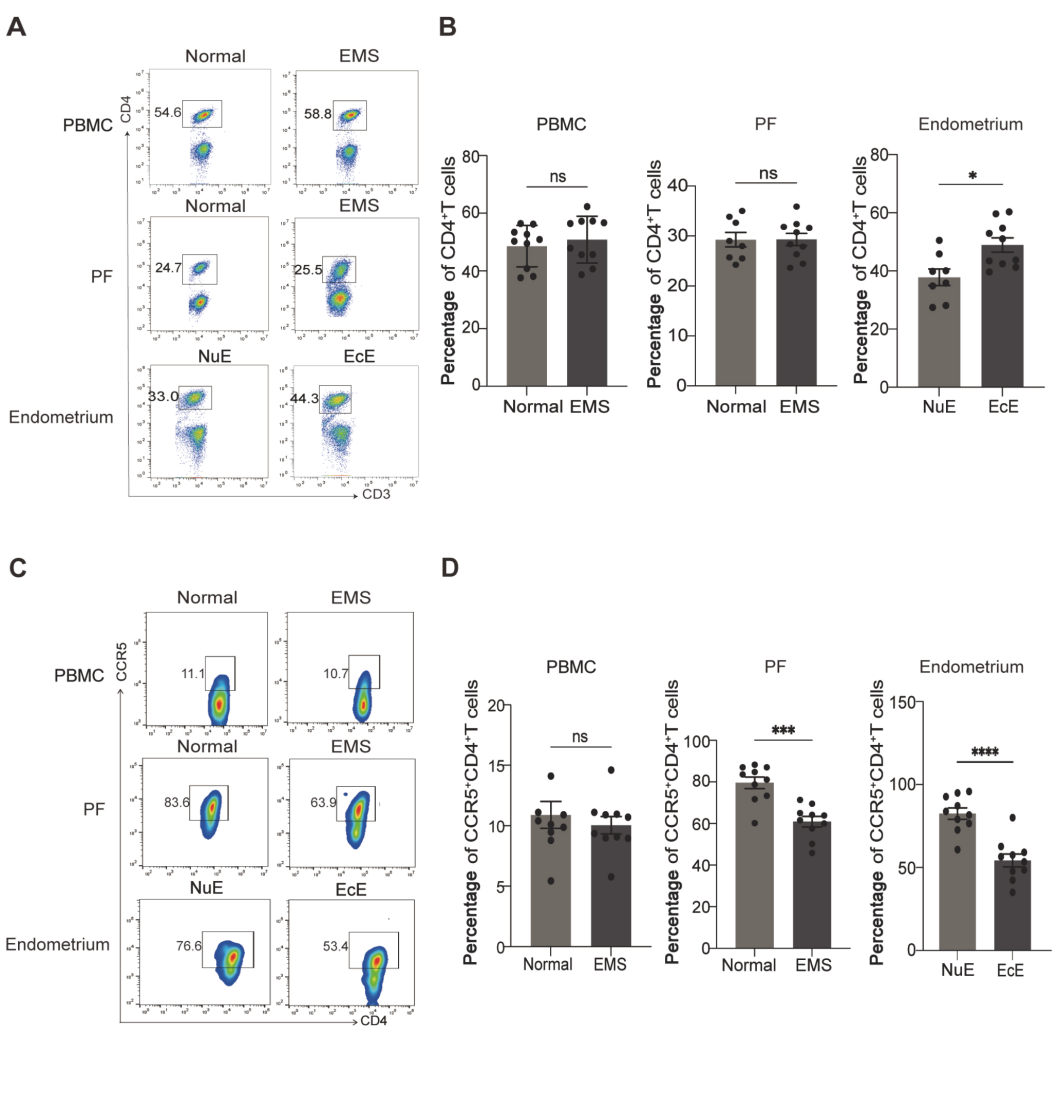
Figure 1 Reduced infiltration of CCR5 ^+^CD4 ^+^ T cells in ectopic lesions from patients with EMS (A,B) Flow cytometry was used to determine the proportions of CD4+ T cells in the peripheral blood mononuclear cells (PBMCs), PF, eutopic endometria of normal patients (NuE), and ectopic endometria of EMS patients (EcE). (C,D) Flow cytometry was used to determine the proportions of CCR5+CD4+ T cells in PBMCs, PFs, NuE, and EcE. *P < 0.05, ***P < 0.001. ns, not significant.

Flow cytometry experiments were conducted to evaluate the changes in peripheral and peritoneal total CD4 ^+^ T cells in EMS. The results revealed no significant difference in peripheral or peritoneal CD4 ^+^ T cells between EMS patients and healthy controls. In contrast, the proportion of tissual CD4 ^+^ T cells was obviously greater in the ectopic lesions than in the eutopic endometrium ( Figure 1A,B).

Next, we focused on the changes in CCR5 ^+^CD4 ^+^ T-cell subsets between EMS patients and healthy controls. The proportion of peripheral CCR5 ^+^CD4 ^+^ T cells from EMS patients was not significantly different from that from healthy controls ( Figure 1C,D). Compared with those from normal controls, CCR5 ^+^CD4 ^+^ T cells derived from PF and ectopic lesions were markedly decreased ( Figure 1C,D), suggesting the potential role of CCR5 ^+^CD4 ^+^ T cells in the development of EMS.

### Impaired IFN-γ production by CCR5 ^+^CD4 ^+^ T cells in patients with EMS

**Figure 2:**
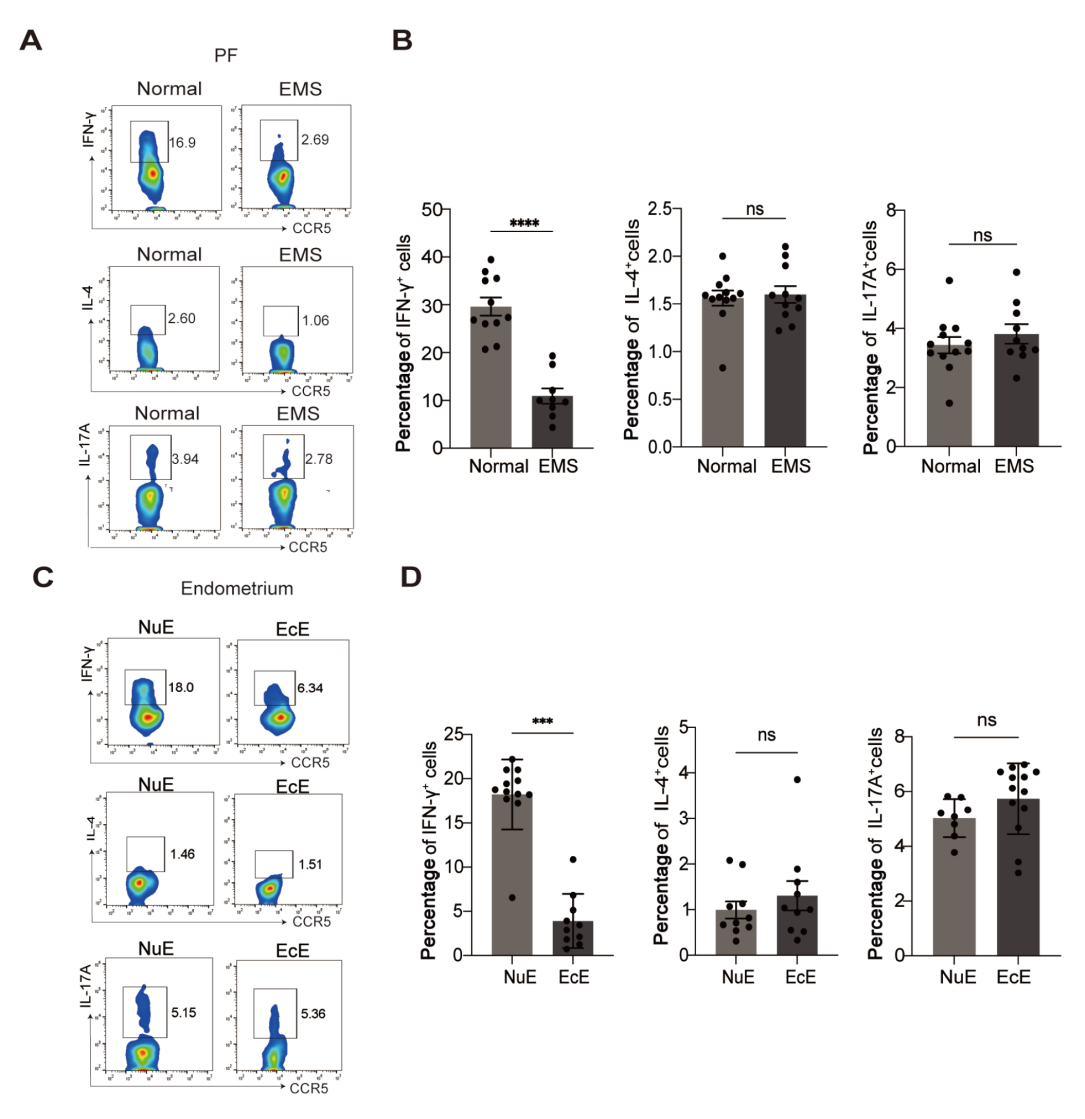
Figure 2 Impaired IFN-γ production of CCR5 ^+^CD4 ^+^ T cells in patients with EMS (A,B) The expressions of IFN-γ, IL-4 and IL-17A in CCR5+CD4+ T cells in the PF of normal patients and EMS patients were detected by flow cytometry. (C,D) The expressions of IFN-γ, IL-4 and IL-17A in CCR5+CD4+ T cells in NuE and EcE were detected by flow cytometry. ***P < 0.001, ****P < 0.0001. ns, not significant.

The immunoregulatory function of effector T cells mainly depends on their cytokine production. Hence, we detected the cytokine profile of CCR5 ^+^CD4 ^+^ T cells by FACS. As shown in Figure 2A,B, the production of the Th1 cytokine IFN-γ in the peritoneal CCR5 ^+^CD4 ^+^ T cells of EMS patients was notably lower than that in the control group. Furthermore, impaired expression of IFN-γ was detected in CCR5 ^+^CD4 ^+^ T cells derived from ectopic lesions compared with those derived from the control group ( Figure 2C,D). However, no significant difference in IL-4 (Th2 cytokine) or IL-17A (Th17 cytokine) expression was detected in CCR5 ^+^CD4 ^+^ T cells derived from the PF ( Figure 2A,B) or ectopic endometrium ( Figure 2C,D) between EMS patients and healthy women. These results suggested that the local decrease in the proportion of CCR5 ^+^CD4 ^+^ T cells was accompanied by impaired IFN-γ production.

### Differential gene expression profiles between peripheral CCR5 ^+^CD4 ^+^ T cells and CCR5 ^‒^CD4 ^+^ T cells

**Figure 3:**
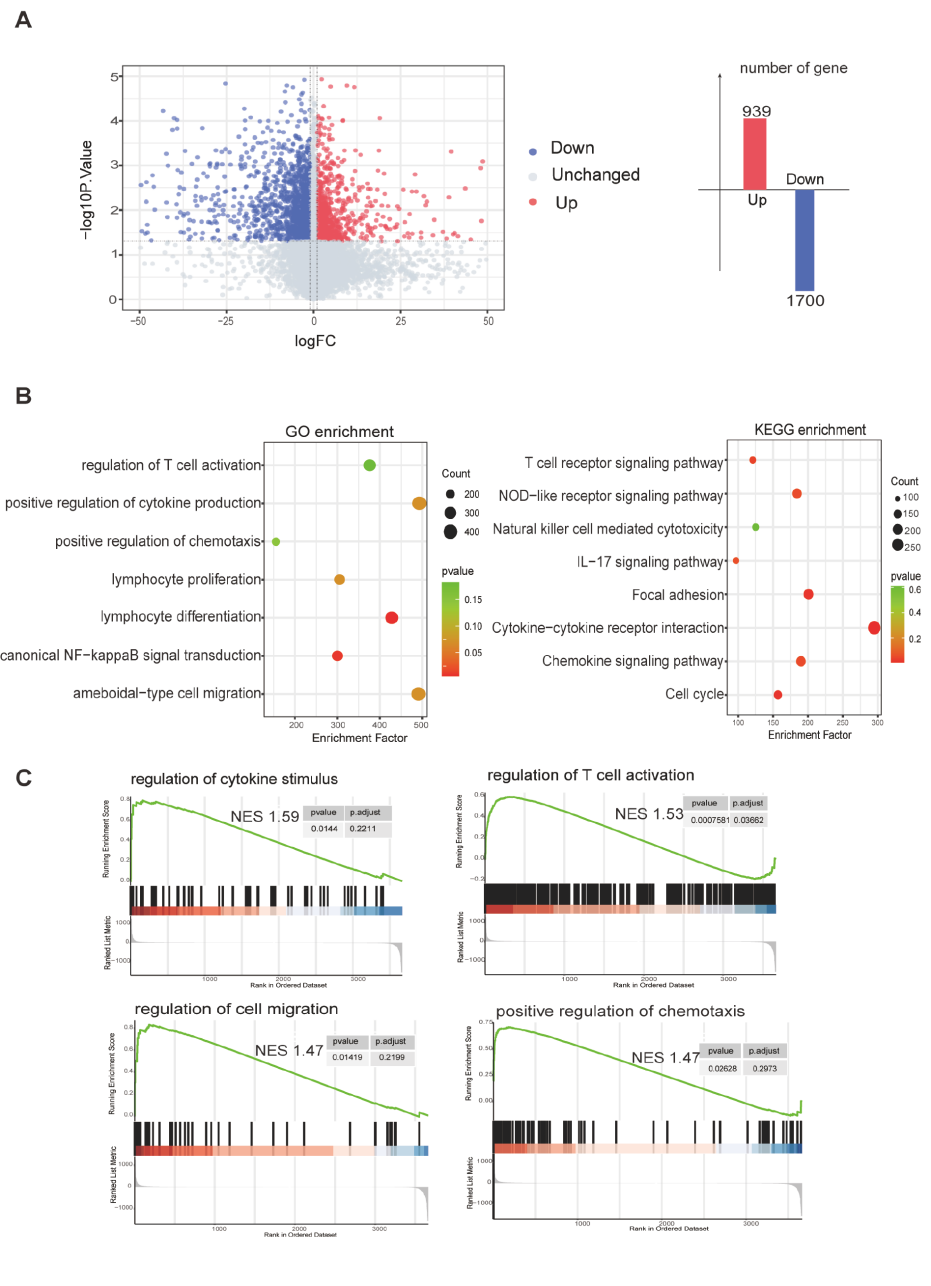
Figure 3 Differential gene expression profiles between peripheral CCR5 ^+^CD4 ^+^ T cells and CCR5 ^–^CD4 ^+^ T cells (A) Volcano map of DEGs identified via RNA-seq in CCR5+CD4+ T cells and CCR5–CD4+ T cells. (B) GO and KEGG analyses of the DEGs identified via RNA-seq in CCR5+CD4+ T cells and CCR5–CD4+ T cells. (C) GSEA of the DEGs identified via RNA-seq in CCR5+CD4+ T cells and CCR5–CD4+ T cells.

To investigate the signatures of gene expression in CCR5 ^+^CD4 ^+^ T cells, we performed RNA-seq. Peripheral CCR5 ^+^CD4 ^+^ T cells and CCR5 ^‒^CD4 ^+^ T cells were collected from 3 normal patients via microbead sorting. A total of 2639 differentially expressed genes (DEGs) were identified in CCR5 ^+^CD4 ^+^ T cells compared with CCR5 ^‒^CD4 ^+^ T cells, including 939 upregulated genes and 1700 downregulated genes ( Figure 3A). GO and KEGG analyses revealed that the DEGs were related mainly to T-cell activation, cytokine production, stimulus of chemotaxis, and cytotoxicity ( Figure 3B). GSEA further confirmed that CCR5 ^+^CD4 ^+^ T cells, but not CCR5 ^‒^CD4 ^+^ T cells, displayed a dominant function in regulating cytokine production, activation, and migration of immune cells ( Figure 3C).

### Decreased infiltration of CCR5 ^+^CD4 ^+^ T cells in ectopic lesions because of deficient production of CCL5 from ecESCs

**Figure 4:**
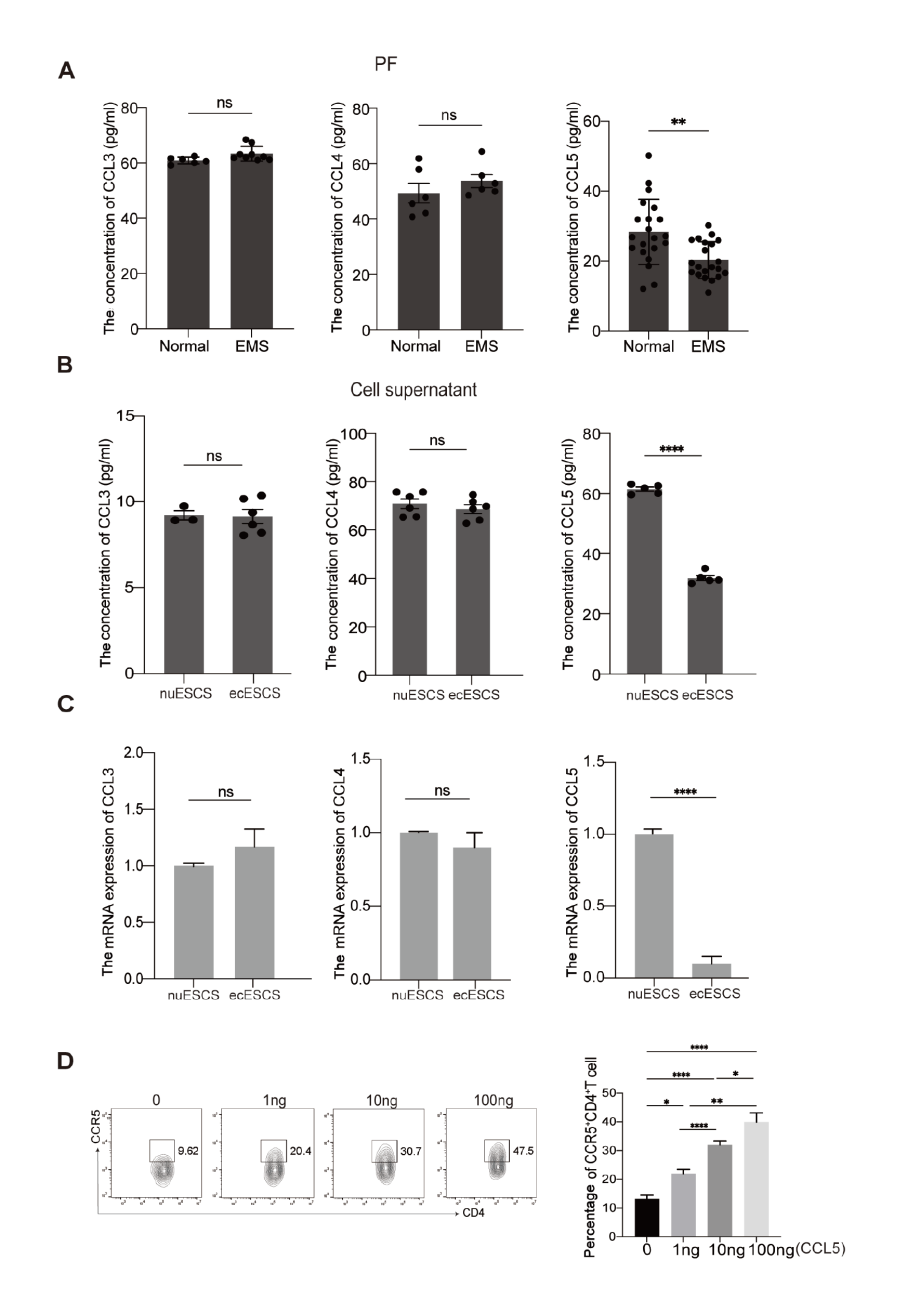
Figure 4 Decreased infiltration of CCR5 ^+^CD4 ^+^ T cells in ectopic lesions because of deficient production of CCL5 from ecESCs (A) ELISA was used to detect CCL3, CCL4, and CCL5 concentrations in the PF of normal and EMS patients. (B) The concentrations of CCL3, CCL4, and CCL5 in the cell supernatants of eutopic endometrial stromal cells from normal patients (nuESCs) and ectopic endometrial stromal cells from EMS patients (ecESCs) were detected via ELISA kits. (C) The transcription levels of CCL3, CCL4 and CCL5 in nuESCs and ecESCs were detected by RT-PCR. (D) Different concentrations of CCL5 (0, 1, 10, or 100 ng) were cocultured with PBMCs from normal women for 4 h, and the proportion of CCR5+CD4+ T cells in the chamber was detected by flow cytometry. *P < 0.05, **P < 0.01, ****P < 0.0001. ns, not significant.

The reason why CCR5 ^+^CD4 ^+^ T cells fail to adequately infiltrate ectopic lesions was further explored. RT-PCR and ELISA were used to evaluate the mRNA and protein levels of CCL3, CCL4, and CCL5 (ligands for CCR5) in ecESCs, which account for the largest number of cells in the ectopic lesion. As shown in Figure 4A, the production of CCL5 was decreased in the PF of EMS patients compared with that in the control group. Primary ectopic and eutopic ESCs were isolated and cultured, and the supernatants were collected after 48 h. A notable reduction in CCL5 was detected in the supernatant of ecESCs ( Figure 4B). However, the concentrations of CCL3 and CCL4 in the PF and cell supernatants did not differ between the two groups ( Figure 4A,B). The RT-PCR results confirmed the conclusion above ( Figure 4C). Next, we observed the chemotactic impact of CCL5 on CCR5 ^+^CD4 ^+^ T cells through cell chemotaxis experiments. The results demonstrated that CCL5 is able to recruit CCR5 ^+^CD4 ^+^ T cells in a dose-dependent manner ( Figure 4D). These findings indicate that a lack of CCL5 deficiency in ecESCs may impede the infiltration of CCR5 ^+^CD4 ^+^ T cells into the ectopic lesions of EMS patients.

### Impaired infiltration of CCR5 ^+^CD4 ^+^ T cells in ectopic lesions of EMS mice

**Figure 5:**
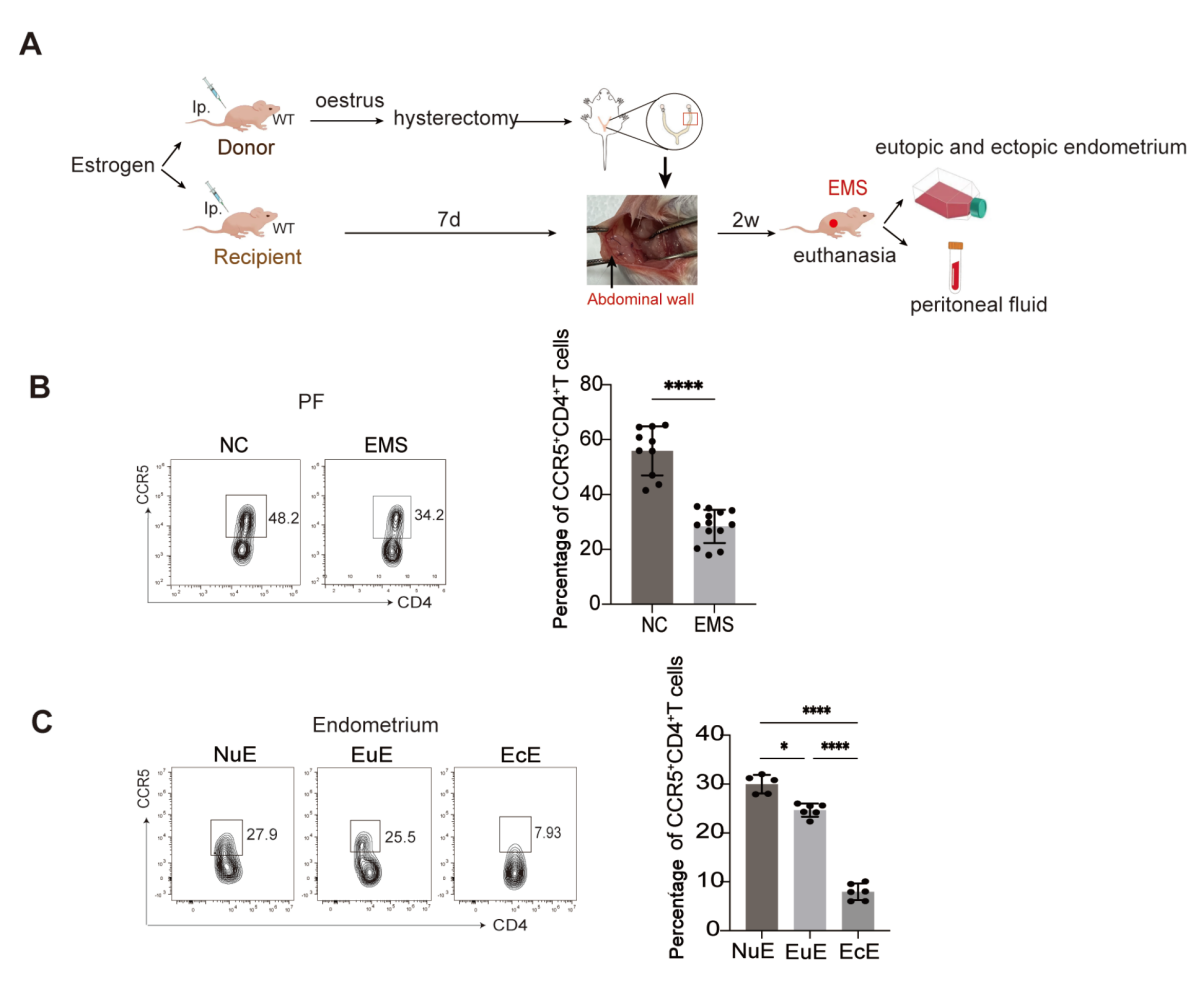
Figure 5 Impaired infiltration of CCR5 ^+^CD4 ^+^ T cells in the ectopic lesions of EMS mice (A) Flow chart of the EMS mouse model. (B) Flow cytometry was used to determine the proportion of CCR5+CD4+ T cells in the PF of normal mice (NC) and EMS mice. (C) Flow cytometry was used to determine the proportion of CCR5+CD4+ T cells in the eutopic endometria of normal mice (NuE), the eutopic endometria of EMS mice (EuE), and the ectopic endometria of EMS mice (EcE). *P < 0.05, ****P < 0.0001.

We established EMS models using wild-type C57BL/6J mice according to the flow diagram ( Figure 5A). Consistent with the above results observed in the PF of EMS patients, the number of peritoneal CCR5 ^+^CD4 ^+^ T cells was obviously lower in the EMS group than in the control group ( Figure 5B). The proportion of CCR5 ^+^CD4 ^+^ T cells collected from ectopic lesions was also lower than that in the eutopic endometria of control mice ( Figure 5C), suggesting attenuated infiltration of CCR5 ^+^CD4 ^+^ T cells.

### Acceleration of EMS growth in the *CCL5*-KO endometrium is accompanied by deficient recruitment of CCR5 ^+^CD4 ^+^ T cells

**Figure 6:**
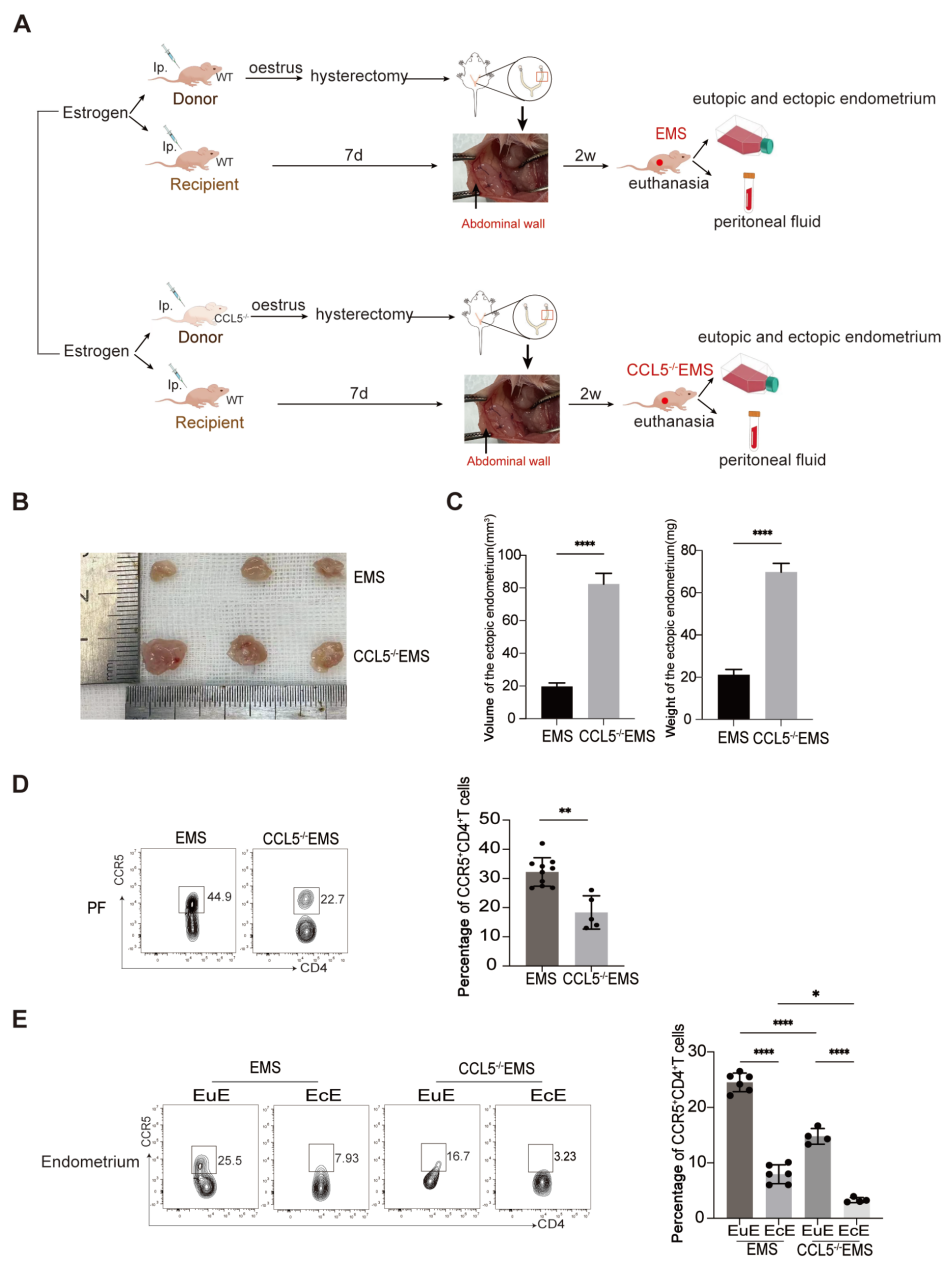
Figure 6 Acceleration of EMS growth by the CCL5-KO endometrium is accompanied by deficient recruitment of CCR5 ^+^CD4 ^+^ T cells (A) Flow chart of model EMS mice and CCL5–/– EMS mice. (B,C) The size and weight of endometriosis lesions. (D) Flow cytometry was used to determine the proportion of CCR5+CD4+ T cells in the PF of EMS mice and CCL5–/– EMS mice. (E) Flow cytometry was used to determine the proportions of CCR5+ CD4+ T cells in the EuE and EcE of EMS mice and CCL5–/– EMS mice. *P < 0.05, **P < 0.01, ****P < 0.0001.

To verify the effects of CCL5 deficiency in the endometrium on the pathogenesis of EMS and recruitment of CCR5 ^+^CD4 ^+^ T cells, the endometrium was stripped from wild-type (WT) and CCL5 ^–/–^ mice as donors, and the stripped endometrium was separately sutured to the peritoneum of the recipient WT mice, as shown in Figure 6A. Compared with those in the WT-EMS group, the size and weight of the ectopic lesions in the CCL5 ^–/–^-EMS group were significantly greater ( Figure 6B,C). We also detected a notable reduction in the proportion of peritoneal CCR5 ^+^CD4 ^+^ T cells in the CCL5 ^–/–^-EMS group compared with the control group ( Figure 6D). Furthermore, impaired infiltration of CCR5 ^+^CD4 ^+^ T cells was observed in the ectopic lesions of CCL5 ^–/–^-EMS mice ( Figure 6E). *In vivo* experiments directly verified that CCL5 deficiency in ectopic lesions could lead to EMS progression and reduce the accumulation of CCR5 ^+^CD4 ^+^ T cells in ectopic lesions.

## Discussion

Retrograde menstruation is the most accepted theory to explain the development of EMS. However, the theory of retrograde menstruation alone cannot fully explain the pathogenesis of EMS. Currently, other theories, including hormonal imbalance between estrogen and progesterone, an abdominal inflammatory response, and a dysregulated immune microenvironment, are also considered to contribute to the development of EMS. Immune microenvironment dysfunction has been proposed as a critical facilitator of endometriotic lesion growth after retrograde menstruation [22].

Peritoneal immune cells, including macrophages [ 8, 23] , neutrophils [ 24, 25] , natural killer cells [ 26, 27] , dendritic cells [28], B cells [ 29– 31] , and CD8 ^+^ T cells [32] reportedly take part in the progression of EMS by regulating cytokine production, angiogenesis, and the inflammatory response. CD4 ^+^ T cells can regulate these cells in various ways [ 33– 38] ; thus, CD4 ^+^ T cells may play an important role in the occurrence and development of EMS. However, few studies have investigated the specific molecular mechanism by which CD4 ^+^ T cells participate in EMS. CD4 ^+^ T cells may regulate the occurrence and development of heterotopic endometrial cells by participating in ectopic endometrial cell adhesion, proliferation, invasion, the inflammatory response, and angiogenesis [ 10, 11] . Previous studies reported that T regulatory cells (Tregs) are more abundant in the peritoneal fluid of patients with EMS, which promotes the local formation of an immune tolerance microenvironment in the ectopic endometrium [ 39– 41] . Gogacz *et al*. [42] reported that the proportion of Th17 cells is associated with the severity of EMS. IL-17 produced by Th17 cells can stimulate the expression of angiogenic factors and proinflammatory cytokines and accelerate the progression of EMS [43]. Borthwick *et al*. [19] reported that IL-4 secreted by Th2 cells could induce fibroblast differentiation into myofibroblasts, which is beneficial for the development of fibrosis in the ectopic endometrium. There are few reports about Th1 cells in EMS, but Podgaec *et al*. [44] reported that the Th1 cell response pattern is related to the severity of EMS and that the production of proinflammatory factors by Th1 cells leads to the growth of ectopic lesions in the advanced stage of the disease.

Therefore, is there a specific EMS-associated CD4 ^+^ T-cell subpopulation whose abnormal local accumulation or recruitment disorders can directly affect EMS pathogenesis? Compared with CCR5 ^–^ T cells, CCR5 ^+^ T cells have been shown to have a greater ability to migrate to tumor sites [ 45, 46] CCR5 ^+^CD4 ^+^ T cells are necessary for the adaptive immune response in tumors. They can activate CD8 ^+^ T cells, induce the maturation of DCs, and increase the activity of APCs, thus exerting an antitumor effect [21]. However, whether CCR5 ^+^CD4 ^+^ T cells play a role in EMS development has not been studied.

In this study, we found that the proportions of CD4 ^+^ T cells in the PBMCs and PFs of EMS patients were not different from those of normal controls. However, the proportion of CD4 ^+^ T cells in the EcE group significantly increased. Compared with normal controls, EMS patients presented insufficient enrichment of CCR5 ^+^CD4 ^+^ T cells accompanied by impaired expression of IFN-γ in the PF and EcE. We performed RNA-seq on CCR5 ^-^CD4 ^+^ T cells and CCR5 ^+^CD4 ^+^T cells. The enrichment analysis revealed that CCR5 ^–^ T cells and CCR5 ^+^ T cells presented significant differences in T-cell activation, cytokine production, chemotaxis, and cytotoxicity.

ecESCs account for the largest number of cells in ectopic lesions. It has been reported that ecESCs can regulate peritoneal immune cells by secreting proinflammatory cytokines, chemokines, and proangiogenesis cytokines to promote self-proliferation and invasion, eventually leading to EMS [ 47– 49] . We detected the concentrations of CCL3, CCL4, and CCL5 (ligands of CCR5) in the PF of patients and in the cell supernatants of ecESCs. We found that CCL5 expression in EMS patients (stage III–IV) was significantly lower than that in control patients, but there was no difference in the concentrations of CCL3 and CCL4 between normal patients and EMS patients. The pelvic and peritoneal microenvironments of EMS are not dominated by purely proinflammatory factors but rather a mixed pattern of proinflammatory and anti-inflammatory cytokines. The levels of proinflammatory cytokines in the PF of EMS patients increased significantly in the early stage (stage I–II) [ 50– 52] , whereas the levels of anti-inflammatory cytokines increased mainly in the late stage (stage III–IV) [ 47, 53– 56] . The expressions of the accompanying chemokines increased significantly in the early stage of the disease but tended to decrease in the late stage of the disease. The EMS samples were in the late stage, CCL5 may have been depleted, and its expression may have decreased in the late stage. The results of the mouse experiments revealed that insufficient infiltration of CCR5 ^+^CD4 ^+^ T cells also existed in the PF and EcE of EMS mice. Compared with those from WT mice, the endometria from CCL5 ^–/–^ mice were more likely to generate ectopic lesions in the peritoneum of recipient mice and recruit fewer CCR5 ^+^CD4 ^+^ T cells. Therefore, the disordered local infiltration of CCR5 ^+^CD4 ^+^ T cells may be caused by the decreased ability of ecESCs to secrete CCL5.

In summary, our research revealed that decreased CCR5 ^+^CD4 ^+^ T cells and their impaired ability to produce IFN-γ are present in the ectopic lesions of EMS patients, which results in reduced production of CCL5 in ecESCs. These data indicate that deficient expression of CCL5 and impaired CCR5 ^+^CD4 ^+^ T cells in ectopic lesions may be potential causes of EMS development.
